# Lamivudine improves cognitive decline in SAMP8 mice: Integrating in vivo pharmacological evaluation and network pharmacology

**DOI:** 10.1111/jcmm.16811

**Published:** 2021-08-10

**Authors:** Ming Li, Jie Zhao, Qi Tang, Qingchen Zhang, Yong Wang, Jian Zhang, Yingying Hao, Xiaohui Bai, Zhiming Lu

**Affiliations:** ^1^ Department of Clinical Laboratory Shandong Provincial Hospital Cheeloo College of Medicine Shandong University Jinan China; ^2^ Department of Clinical Laboratory Shandong Provincial Hospital Affiliated to Shandong First Medical University Jinan China; ^3^ Department of Radiology Jinan Central Hospital, Cheeloo College of Medicine, Shandong University Jinan China

**Keywords:** ageing, cognitive ability, lamivudine, long interspersed element‐1, neurodegenerative diseases

## Abstract

The reverse transcriptase inhibitors such as lamivudine (3TC) play important roles in anti‐ageing, but their effects on neurodegenerative diseases caused by ageing are not clear, especially on the functions of the nervous system such as cognition. In this study, we administered 3TC to senescence‐accelerated mouse prone 8 (SAMP8) mice by gastric perfusion (100 mg/kg) for 4 weeks. Our results showed that 3TC significantly improved the ageing status of SAMP8 mice, especially the decline of cognitive ability evaluated by the Morris water maze test. To further investigate the molecular mechanisms of improving the ageing status of SAMP8 mice by 3TC, the qPCR and tissue staining methods were used to study the brain tissues (i.e., hippocampus and cortex) of mice, while the network pharmacology analysis was applied to investigate the potential targets of 3TC. The results showed that the mRNA levels of genes related to long interspersed element‐1, type 1 interferon response, the senescence‐associated secretion phenotype and the Alzheimer's disease in the hippocampus and cortex of SAMP8 mice were increased due to senescence, but this trend was reversed partially by 3TC. Results of histological studies showed that 3TC reduced the death of hippocampal neurons, while the results of network pharmacology analysis indicated that 3TC may exert its influence through multiple pathways, including the oestrogen signalling and the PI3K/Akt and neuroactive ligand‐receptor interaction signalling pathways, which we have verified through in vitro experiments. These findings provide evidence for the therapeutic potential of 3TC in the treatment of neurodegenerative diseases.

## 1 INTRODUCTION

To date, ageing and neurodegenerative diseases, such as Alzheimer's (AD) or Parkinson's diseases linked to ageing, are ever‐increasing due to the global demographic changes.^1^, ^2^ The cognitive decline represents a serious hindrance to achieving a long and healthy life. For decades, there have been considerable efforts devoted to searching for medications that can be used to treat cognitive degeneration with ageing.^3^ In recent years, the long interspersed element‐1 (L1) has gained increasing attention as a potential novel target for studying senescence.^4^ The L1 belongs to the most abundant family of non‐long terminal repeat retrotransposons with ~500,000 copies, comprising ~17% of the human genome. The L1 gene comprises an operon with two open reading frames (ORF1 and ORF2), both of which are required for retrotransposition.^5^, ^6^, ^7^ The ORF1 encodes a protein (ORF1p) with reverse transcriptase activities which can be inhibited by nucleoside analogues, such as lamivudine (2’‐3’‐deoxy‐3’‐thiocytidine, 3TC).^8^

Active L1 can synthesize cDNA outside the chromatin and then reinsert it into the genome catalysed by ORF1p, ultimately altering the expression of other genes and resulting in diseases associated with genomic instability.^9^, ^10^ The host generally inhibits the transposable activity of L1 through a series of regulations, such as epigenetic modification and host limiting factor regulation.^11^, ^12^ Transposition of L1 generally occurs only in either germ or cancer cells, while recent studies have shown that L1 is also highly active in the central nervous system or ageing cells, though the underlying neural mechanisms of L1 are unclear.^13^

Studies have shown that factors (e.g., TREX1, RB1 and FOXA1) related to the monitoring mechanism of L1 are dysfunctional in cell senescence, resulting in the activation of L1. TREX1 is a 3’ exonuclease that degrades foreign invading DNAs and its loss has been associated with the accumulation of cytoplasmic L1 cDNA.^14^ RB1 has been shown to bind to repetitive elements, including L1 elements, and promote their heterochromatinization.^15^ And FOXA1 is up‐regulated in senescent cells13 and bound to the central region of the L1 5’ UTR. Activated L1 further activates type‐I interferon (IFN‐I) response, which induces inflammation through reverse transcriptional cDNA and maintains the senescence‐associated secretion phenotype (SASP). Therefore, as an important factor in aseptic inflammation caused by ageing, L1 is considered as an important target for the treatment of ageing‐related diseases. In the elderly mice treated with 3TC, the accumulation of inflammatory factors is improved by inhibiting extracellular transcription of L1 in muscular and other tissues.^16^ To date, the studies of the effects of reverse transcriptase inhibitors on the functions of the nervous system are sparse.

Recently, thousands of variants of genomic cDNA (gencDNA) have been found in the brains of deceased patients with AD, resulting from the re‐insertion of characteristic RNA splicing variants into the genome.^17^ This phenomenon is considered as an important cause of AD. The factors causing the generation of gencDNAs include transcription, DNA destruction, reverse transcriptase and ageing.^18^ Studies have demonstrated that the process of gencDNA formation is blocked by using the nucleoside reverse transcriptase inhibitors in Chinese hamster ovary (CHO) cell lines with endogenous reverse transcriptase activities.^19^ Furthermore, AIDS patients over the age of 65 who have been taking reverse transcriptase inhibitors for a long time rarely developed AD.^20^ Therefore, we speculate that 3TC, as a reverse transcriptase inhibitor, may be a potential drug for the treatment of cognitive impairment and neurodegenerative diseases caused by ageing.

It was shown that senescence‐accelerated prone 8 (SAMP8) mice, as a model for studying human ageing and age‐related diseases, display many features known to occur in the early stages of neurodegenerative diseases, such as increased oxidative stress, neuroinflammation and cognitive decline.^21^ In this study, therefore, SAMP8 mice were selected as an ideal animal model for neurodegenerative diseases caused by ageing. We focused on the cognitive effects of 3TC on premature ageing mice and the molecular pathways associated with ageing, providing evidence for the role of reverse transcriptase inhibitors in the treatment of neurodegenerative diseases due to ageing. We further applied the network pharmacology method to predict the targets of 3TC, construct networks and analyse the biological functions and pathways related to 3TC.

## 2 EXPERIMENTAL SECTION

### 2.1 Animals

**FIGURE 1:**
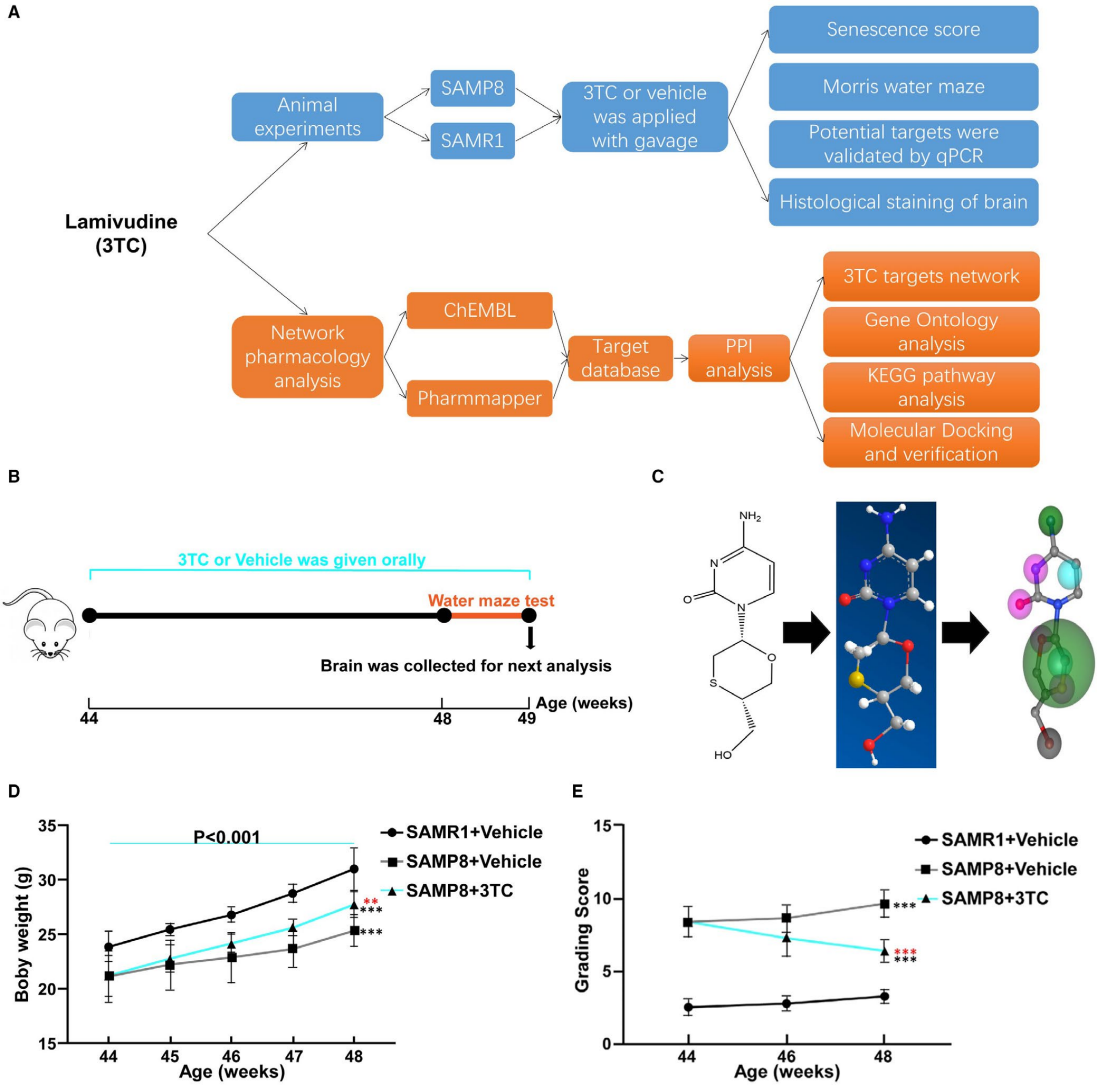
Experimental design and the effects of 3TC on improving relative weight loss due to ageing and senescence grading scores. (A) Flow chart of the pharmacological experiments to study the effect of 3TC on the cognitive ability of SAMP8 mice. (B) Schematic presentation of the animal experiment schedule. (C) The conceptual workflow for predicting the drug‐target interactions using Pharmmapper showing, from left to right, the molecular structure of 3TC, the 3D structural diagram of 3TC and the pharmacophore model predicted with 3TC binding to the oestrogen receptor with the highest normalized fit scores. (D) The growth pattern of body weight in each group of mice. (E) Changes in grading scores before and after the treatment of 3TC. SAMR1+Vehicle: 44‐week‐old SAMR1 mice vehicle‐treated for 4 weeks. SAMP8+Vehicle: 44‐week‐old SAMP8 mice vehicle‐treated for 4 weeks. SAMP8+3TC: 44‐week‐old SAMP8 mice 3TC‐treated for 4 weeks. Student's t‐test: *p < 0.05, **p < 0.01 and ***p < 0.001. ns: no statistical significance. Black asterisks represent comparison with the SAMR1+vehicle group. Red asterisks represent comparison with the SAMP8+vehicle group. The blue line marks the SAMP8+3TC group at the age of 44 and 48 weeks

The 44‐week‐old male SAMP8 and cognate normal senescence‐accelerated mouse‐R1 (SAMR1) mice provided by Tianjin University of Traditional Chinese Medicine were used after a 1‐week acclimatization period. The animal experimental protocol was approved by the Animal Ethics Committee of Shandong Provincial Hospital and was performed based on the National Institutes of Health (NIH) Guide for the Care and Use of Laboratory Animals. The mice were kept in autoclaved cages and given sterile food and water. The body weights of the mice were recorded every day. A flow chart of the experiments conducted in this study is given in Figure 1A. Upon completion of the experiments, the mice were euthanized by diethyl ether inhalation.

### 2.2 Preparation and treatment of 3TC

Drugs were freshly prepared on the day of each experiment. The 3TC purchased from MCE (Shanghai, China) was dissolved in DMSO to a final concentration of 2 mmol/L. The stock solution was then diluted to the working concentration using drinking water with the final concentration of DMSO less than 0.1%. Three groups of mice were established: a control group treated with vehicle solution (drinking water), a test group treated with 3TC and the SAMR1 group that received vehicle used as another control group for ageing. The test group orally received 3TC at a dose of 100 mg/kg per day. All mice were fed by intragastric administration for 4 weeks (Figure 1B).

### 2.3 Grading score system

The degree of senescence in the mice was evaluated using a grading score system described previously.^22^ Each category of scores contains five levels, ranging from 0 to 4 with the highest score of the grade indicating the highest degree of the animal's senescence. The scores for each category were assessed separately by four experienced experimenters who were blinded to the treatment conditions (Supplementary Table S1).

### 2.4 Morris water maze test

Morris water maze studies were performed according to Morris’ protocol, including an orientation navigation experiment and probe trials.^23^ The water maze was a circular pool (120 cm in diameter equipped with a platform of 10 cm in diameter) and filled with water at 25 ± 1°C. The mice were trained to find the platform below the surface of the water within one minute. If the mouse did not find the platform within 60 s, it was guided to the platform and was allowed to remain there for 20 s. Each of the mice was trained five times per day with each separated from one another by 15 min. In each trial, the starting position was different, in a pseudorandom order. On the sixth day of the test, the platform was removed and the animal entered the water maze from a point opposite to the quadrant of the maze where the platform used to be during training and it was allowed to explore the maze for 60 s. The swimming path and the amount of time that the mice spent searching for the maze were recorded by the video camera and analysed using EthoVision XT (Noldus Software). Each group consisted of a minimum of eight animals.

### 2.5 RNA extraction and quantitative PCR

Total RNA was extracted from hippocampal and cortical tissues of the mice after the treatments using TRIzol Reagent (Invitrogen) according to the manufacturer's protocol. Each RNA sample (1 μg) was reversely transcribed to generate cDNA using cDNA Reverse Transcription Kit (Takara) and then PCR‐amplified using the TaKaRa Taq Kit (Takara). Primer sets are described in Supplementary Table S2.

### 2.6 Tissue preparation

The animals were anaesthetized with diethyl ether, and their brains were collected following transcardial perfusion with phosphate buffered saline (PBS) and a 4% paraformaldehyde solution. Brain tissues were removed and fixed in paraformaldehyde for over 2 days. The fixed brains were embedded in paraffin with the hippocampal tissue sectioned at a thickness of 5 μm.

### 2.7 Haematoxylin and eosin (H & E) staining

Brain morphology was analysed using haematoxylin and eosin (H & E) staining methods. The paraffin sections of the hippocampal tissues were dewaxed and dehydrated with a gradient of alcohol of a series of concentrations. The HE staining was performed using an H & E staining kit (Solarbio) according to the manufacturer's instructions. After immersed in ethanol and xylene, the sections were mounted with resin and observed under a light microscope (Olympus).

### 2.8 Nissl staining

Nissl staining (Biyuntian) method was used to detect the neuronal injury on the sections of the hippocampal tissues. The sections were incubated with Nissl stain for 30 min and washed with 95% ethanol. The stained cells were counted and photographed under a light microscope (Olympus). Cells were counted on three randomly selected non‐overlapping fields in each slide of the hippocampal tissue with the survival index defined as the number of surviving neurons/total number of neurons.

### 2.9 TUNEL staining

Sections of hippocampal tissues were incubated with proteinase K solution for 15 min followed by incubation with TUNEL reaction mixture (Beyotime). After washing three times with PBS, the sections were mounted with a mounting solution containing the nuclear dye DAPI and observed using either a conventional fluorescence microscope or a confocal laser fluorescence microscope.

### 2.10 Construction of target database of 3TC

The target database of 3TC was constructed based on two databases. First, the potential targets of 3TC were identified using the reverse pharmacodynamic gene map based on the Pharmmapper database (http://www.lilab‐ecust.cn/pharmmapper/results/200816062539.html). All chemical components were converted to the format of ‘.mol2’ by Chembiodraw and uploaded to the Pharmmapper database (Figure 1C). Molecules with Normalized Fit Score larger than 4.0 were identified as potential targets of 3TC. Second, the ChEMBL database (https://www.ebi.ac.uk/chembl/compound_report_card/CHEMBL141/) was used for querying the biological activities of targets or compounds. Proteins verified as derived from *Homo sapiens* were identified as the potential targets of 3TC.

### 2.11 Target protein interaction analysis and network construction

The target proteins screened were integrated for the protein‐protein interaction (PPI) analysis using String 9.1 (http://string‐db.org/) with the protein network interaction diagrams generated. Protein interactions with confidence score >0.4 were selected in designed setting after eliminating duplicates.

To comprehensively investigate the molecular mechanisms of 3TC, the PPI networks were constructed using Cytoscape software version 3.6.0. (http://www.cytoscape.org/). Degree analysis of nodes was performed with the Centiscape 2.2 (http://apps.cytoscape.org/apps/centiscape) to screen hub genes in the networks with the closeness centrality value, betweenness centrality value and degree centrality value set to 0.2, 450 and 50, respectively.

### 2.12 Enriching and screening signalling pathways for 3TC

Gene Ontology (GO) enrichment analysis with biological processes, cellular components and molecular functions of potential targets was carried out for biological function annotation based on a DAVID bioinformatics database (https://david.ncifcrf.gov/gene2gene.jsp). KEGG metabolic pathways were annotated based on the Kyoto Encyclopedia of Genes and Genomes (KEGG) databases using the online KEGG Automatic Annotation Server (http://www.genome.jp/kegg/).

### 2.13 Binding of 3TC to predicted targets

The SDF structure files of compound 3TC were obtained by the pubchem website (https://pubchem.ncbi.nlm.nih.gov/). The SDF file was transformed into a PDB file by OpenBabel2.3.2 software, and the receptor proteins ADRB2 (PDBID:3KJ6), AKT1 (PDBID:4EKL) and EGFR (PDBID:6S9C) were obtained from Protein Data Bank database (www.wwpdb.org). The PYMOL2.3.4 software was used to remove water and ligands from the receptor proteins. The docking study was performed using the AutoDock Vina (1.1.2), which is an open‐source molecular docking software developed by Scripps. Tools software was used to modify the four receptor proteins, and the Grid Box command under the Grid program was used to open the Grid Option tool to analyse each receptor protein. The lattice spacing was set to 1, and the centre of the pocket was set as the centre of the binding site.

### 2.14 Cell culture and treatment

Mouse hippocampal neuronal cell lines HT22 was purchased from the Shanghai Cell Biotechnology Company. HT22 cells were cultured in DMEM supplemented with 10% foetal bovine serum (FBS). Cells were seeded in 6‐well plates 1 day prior to experimental treatments. The senescence of cells was induced by formaldehyde (FA), purchased from Sigma‐Aldrich, as previously described.^24^ HT22 cells were divided into three groups, including the FA group treated with 30 µmol/L FA for 24 h, the FA/3TC group treated with both 30 µmol/L FA and 50 µmol/L 3TC, and the control group cultured without either FA or 3TC.

### 2.15 Immunofluorescence and microscopy

The medium was removed, and the cells were fixed using a 2% paraformaldehyde solution for 1 h at room temperature. The immunofluorescence experiment was carried out as previously described.^25^ The antibodies used in this study included the anti‐LINE‐1 ORF1p antibody (1:1000; Sigma‐Aldrich) with Alexa Fluor 594 secondary antibody (1:1000; Invitrogen). The cells were incubated with DAPI (1:500 dilution; Thermo Fisher Scientific) for 1 min to label the nuclei. After the anti‐fluorescence quenching mounting agent was added, the cells were observed using a confocal microscope (Nikon).

### 2.16 Western blot analysis

The expressions of *EGFR*, *p‐AKT1* (Active Akt1: phosphorylated at S473) and *ADRB2* were measured by Western blot analysis. Total protein was collected from the cells after various treatments. Western blot was performed as previously described.^26^ The antibodies used in this study included anti‐EGFR antibody (1:500; Sigma‐Aldrich), anti‐pAKT1 antibody (1:1000; Sigma‐Aldrich) and anti‐ADRB2 antibody (1:200; Sigma‐Aldrich) with anti‐rabbit secondary antibody (1:1,000; Invitrogen). After washing, the blots were developed with the ECL Western blotting detection system (Amersham, Aylesbury, UK).

### 2.17 Data analysis and statistics

Each experiment was repeated at least three times and considered valid with the trials showing similar results. Results were presented as the mean ± standard deviation (SD). The significant difference was determined by either Student's *t*‐test with *p* < 0.05.

## 3 RESULTS

### 3.1 3TC reverses body weight loss caused by ageing and improves the senescence score

The body weight changes of the mice in different experimental groups are shown in Figure 1D. The results showed that all mice gained body weight in 4 weeks. Under standard diet, SAMP8 mice treated with 3TC gained weight faster than those treated with vehicle. The results of the senescence scores before and after the treatments of 3TC or vehicle showed that the average senescence scores of SAMP8 mice were relatively higher, while the reversed trend was achieved by 3TC treatment (Figure 1E). The results of the senescence‐related scores of each group of mice collected on the last day of the experiment showed that SAMP8 mice treated with 3TC showed significant improvements in several senescence scores, including the glossiness of skin (*p* < 0.01, *t* = 3.901), the corneal opacity (*p* < 0.01, *t* = 4.753) and the ulcer of the cornea (*p* < 0.05, *t* = 6.125) (Supplementary Table S1).

### 3.2 3TC improves the cognitive decline of SAMP8 mice

**FIGURE 2:**
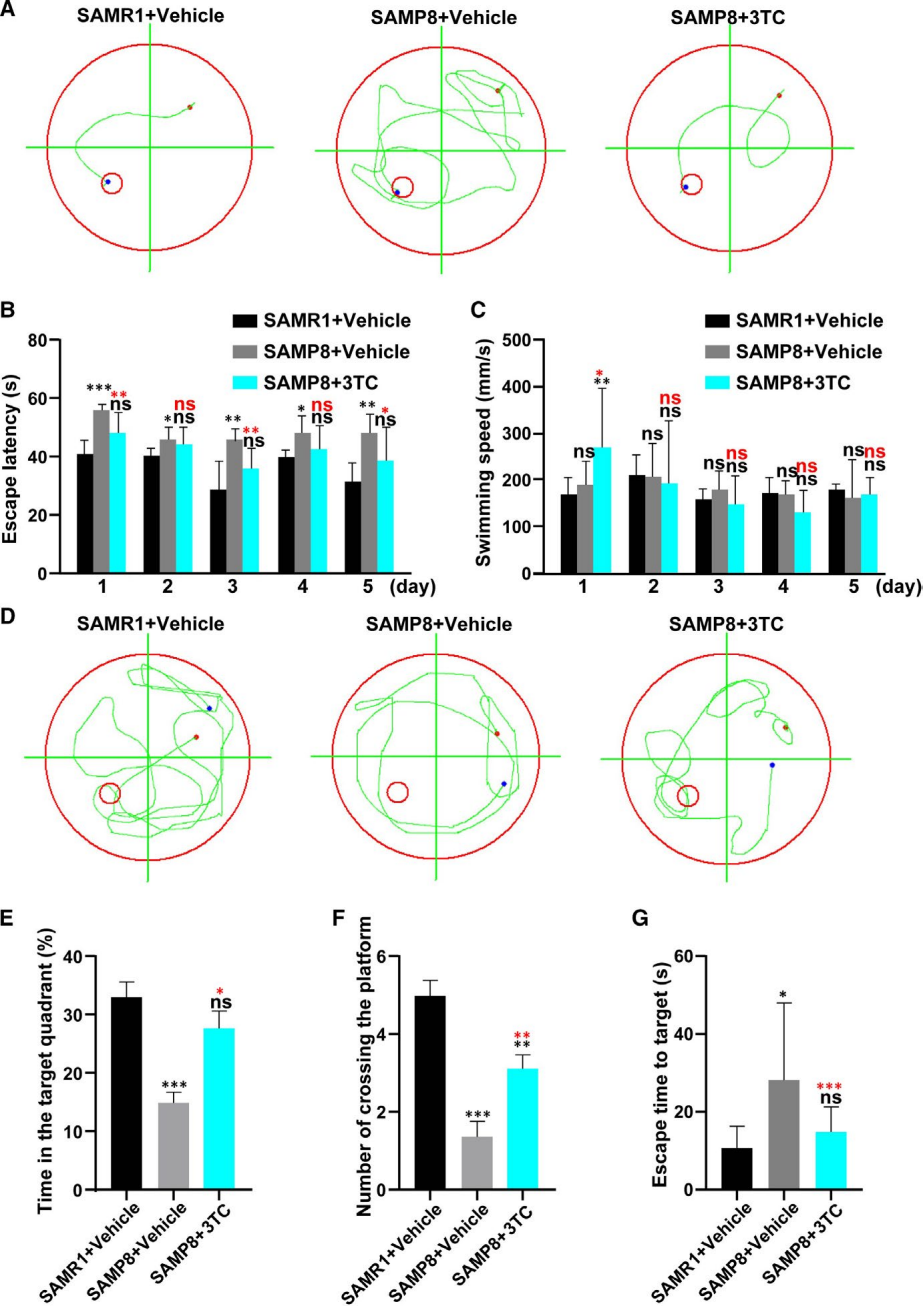
The effects of 3TC treatment on improvements of the cognitive decline in SAMP8 mice. (A) Trajectory map of mice after entering the water from the second quadrant during the positioning navigation test on the 5th day. (B) Escape latency in the positioning navigation experiment for each group. (C) Swimming speed of mice in each group in behavioural tests. (D) Trajectory map of mice in the space exploration Experiment. (E) Percentage of total time spent in the target quadrant during the probe trials in each group. (F) The number of times that the mice crossed the platform in each group in the probe trials. (G) Escape time to target. Data are presented as mean ± SD. Student's t‐test: *p < 0.05, **p < 0.01 and ***p < 0.001. ns: no statistical significance. Black asterisks or "ns" represent comparison with the SAMR1+vehicle group. Red asterisks or "ns" represent comparison with the SAMP8+vehicle group. Each group consisted of a minimum of eight animals

The Morris water maze tests were carried out to evaluate the cognitive effects of 3TC (Figure 2). Results of the trajectories and the directional navigation experiments for five consecutive days demonstrated that SAMR1 mice showed significantly lower escape latencies than those of SAMP8 mice (*p* < 0.05, *t* = 3.194) (Figure 2A–B). However, on the third and the fifth days, the escape latencies of the 3TC‐treated SAMP8 mice were significantly shorter in comparison with those vehicle‐treated SAMP8 mice (*p* < 0.05, *t* = 3.115), while no statistical significance was revealed in comparison with the SAMR1 group. Although the swimming speed of SAMP8 mice treated with 3TC on the first day was significantly faster than that of the other two groups, no significant difference was detected among the three experimental groups in the next 4 days of the experiments (Figure 2C).

The results of the probe trials and the trajectory on the 6th day showed that both the SAMR1 and the 3TC‐treated SAMP8 group spent more time in the target quadrant than the vehicle‐treated SAMP8 group (*p* < 0.05, *t* = 4.512; Figure 2D–E). Moreover, the numbers of crossings were also greater in both the SAMR1 mice and the 3TC‐treated SAMP8 group than that in the vehicle‐treated SAMP8 group, with a statistically significant difference revealed between the groups with and without the treatment of 3TC (*p* < 0.05, *t* = 5.79; Figure 2F). The vehicle‐treated SAMP8 group showed the highest latencies to find the platform under the water among all groups with a statistically significant difference revealed between the groups treated and not treated with 3TC (*p* < 0.05, *t* = 4.415; Figure 2G).

### 3.3 Target genes of 3TC revealed by the real‐time quantitative PCR

**FIGURE 3:**
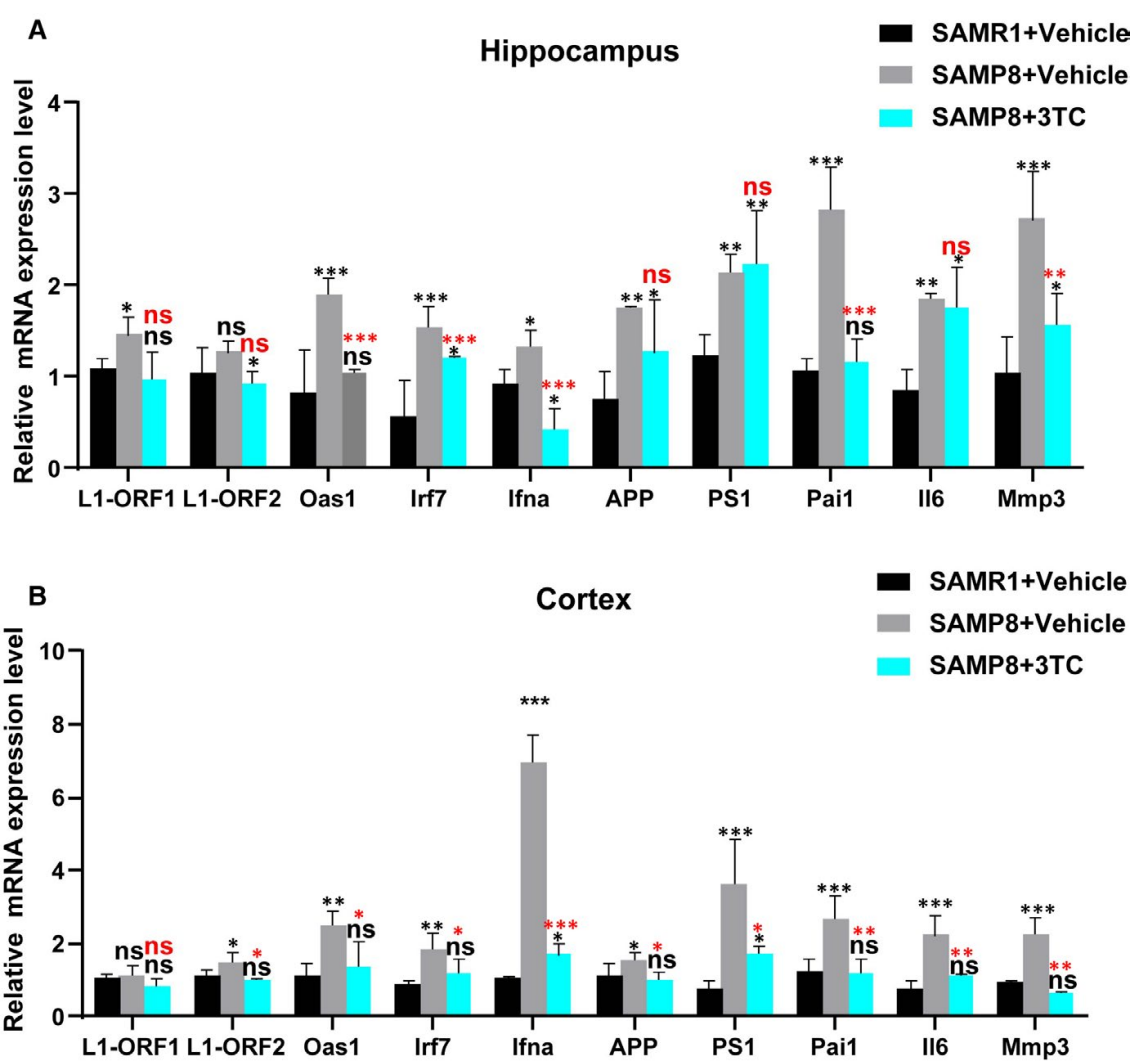
Effects of 3TC treatment on the expressions of candidate genes in mouse brain tissues. Mice were treated with vehicle or 3TC continuously for 4 weeks. For all conditions, the expression of *L1‐ORF1* and *L1‐ORF2*, three representative IFN‐I response genes (*Ifna*, *Irf7* and *Oas1*), two Alzheimer disease‐related genes (*PS‐1* and *APP*) and three representative SASP genes (*Il6*, *Mmp3* and *Pai1*) in the hippocampus (A) and the cortex (B) were assessed by real‐time quantitative PCR with the expression profiling of genes shown in the histogram. Student's *t*‐test: *p < 0.05, **p < 0.01 and ***p < 0.001. ns: no statistical significance. Black asterisks or "ns" represent comparison with the SAMR1+vehicle group. Red asterisks or "ns" represent comparison with the SAMP8+vehicle group

Previous studies have shown that 3TC can reduce the SASP and IFN‐I response signals to the smooth muscle cells of ageing mice, but there is no report on the associated genes of neurological degenerative disease in brain tissue. To explore the effect of 3TC on the mice brain, we investigated the expression of ten candidate genes of interest in the hippocampus and cortex of mice using the real‐time quantitative PCR. In the hippocampus (Figure 3A), the significantly up‐regulated genes for ageing (groups of SAMR1 vs. SAMP8 treated with vehicle) included one transposon‐related gene (*L1‐ORF1*, *p* < 0.05), three IFN‐I response genes (*Ifna*, *Irf7* and *Oas1*, *p* < 0.05), two AD‐related genes (*PS‐1* and *APP*, *p* < 0.01) and three representative SASP genes (*Il6*, *Mmp3* and *Pai1*, *p* < 0.01), while the significantly down‐regulated genes for 3TC treatment (groups of SAMP8 treated with vehicle vs. SAMP8 treated with 3TC) included one transposon‐related gene (*L1‐ORF2*, *p* < 0.05), two IFN‐I response genes (*Ifna* and *Oas1*, *p* < 0.001) and three representative SASP genes (*Il6*, *Mmp3* and *Pai1*, *p* < 0.01). Similar results were obtained in the cortex, though the up‐regulation of the gene related to AD was decreased by the treatment of 3TC in cortex in comparison with the hippocampus (*p* < 0.05; Figure 3B).

### 3.4 3TC attenuates morphological abnormalities and loss of neurons in the hippocampus of SAMP8 mice induced by ageing

**FIGURE 4:**
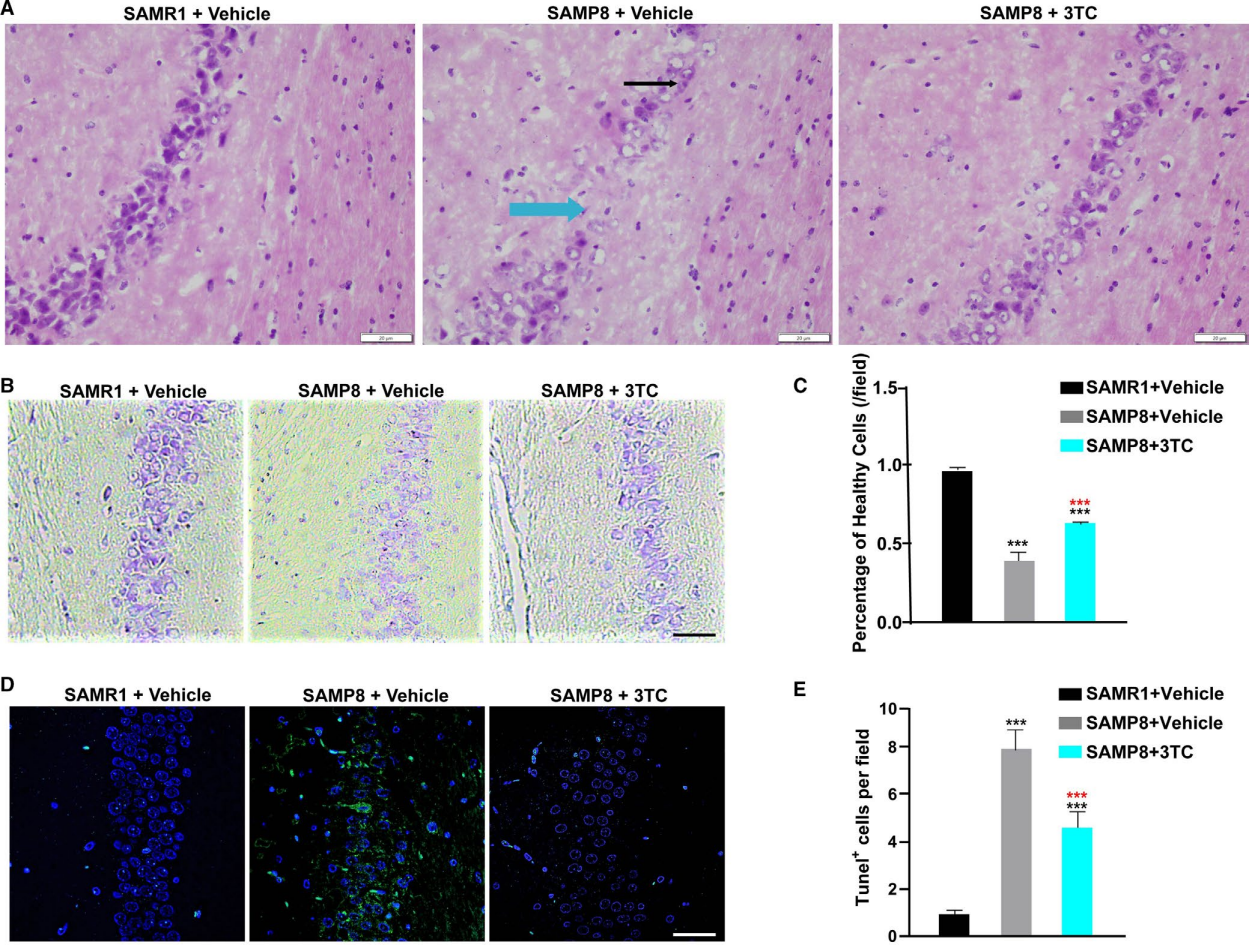
3TC decreases neuronal injury in the hippocampus of SAMP8 mice. (A) Photomicrographs of H & E–stained hippocampus sections for each group of mice (bar = 50 μm). Black arrows mark concentrated nuclei, and blue arrows mark areas where neurons are severely lost and disarranged. (B) Identification of neuronal survival by Nissl staining in the hippocampus (bar = 20 μm). (C) The quantity of healthy neuronal cells in each visual field with densely stained Nissl bodies. (D) Images of apoptotic cells marked by TUNEL (Green) assay in each group of mice (bar = 20 μm). (E) Quantification of TUNEL‐positive cells in each visual field. Data are presented as mean ± SD. Student's *t*‐test: ***p < 0.001. Black asterisks represent comparison with the SAMR1+vehicle group. Red asterisks represent comparison with the SAMP8+vehicle group

We applied both H & E and Nissl staining methods to investigate the morphological changes caused by 3TC in the hippocampus, which was the key area of cognition and memory. In comparison with the group of SAMR1 treated with vehicle, the group of SAMP8 treated with vehicle showed increased clearance rate of hippocampal neurons, slightly disordered arrangement of cells, nuclear concentration found in some neurons and mild hippocampal oedema. These damages were improved in the 3TC‐treated groups (Figure 4A).

The losses of Nissl‐positive viable neuronal cells with typical neuropathological change were observed in SAMP8 group based on Nissl staining, while these changes were partially reversed in the group of SAMP8 treated with 3TC (Figure 4B–C). The neuron death in the hippocampus was also detected by TUNEL staining with the most and the least amounts of TUNEL‐positive cells (green) observed in the group of SAMP8 treated with vehicle and the SAMR1 group treated with vehicle, respectively, while the group of SAMP8 treated with 3TC was revealed significantly less than the group of SAMP8 treated with vehicle (Figure 4D–E).

### 3.5 Putative targets of 3TC

**FIGURE 5:**
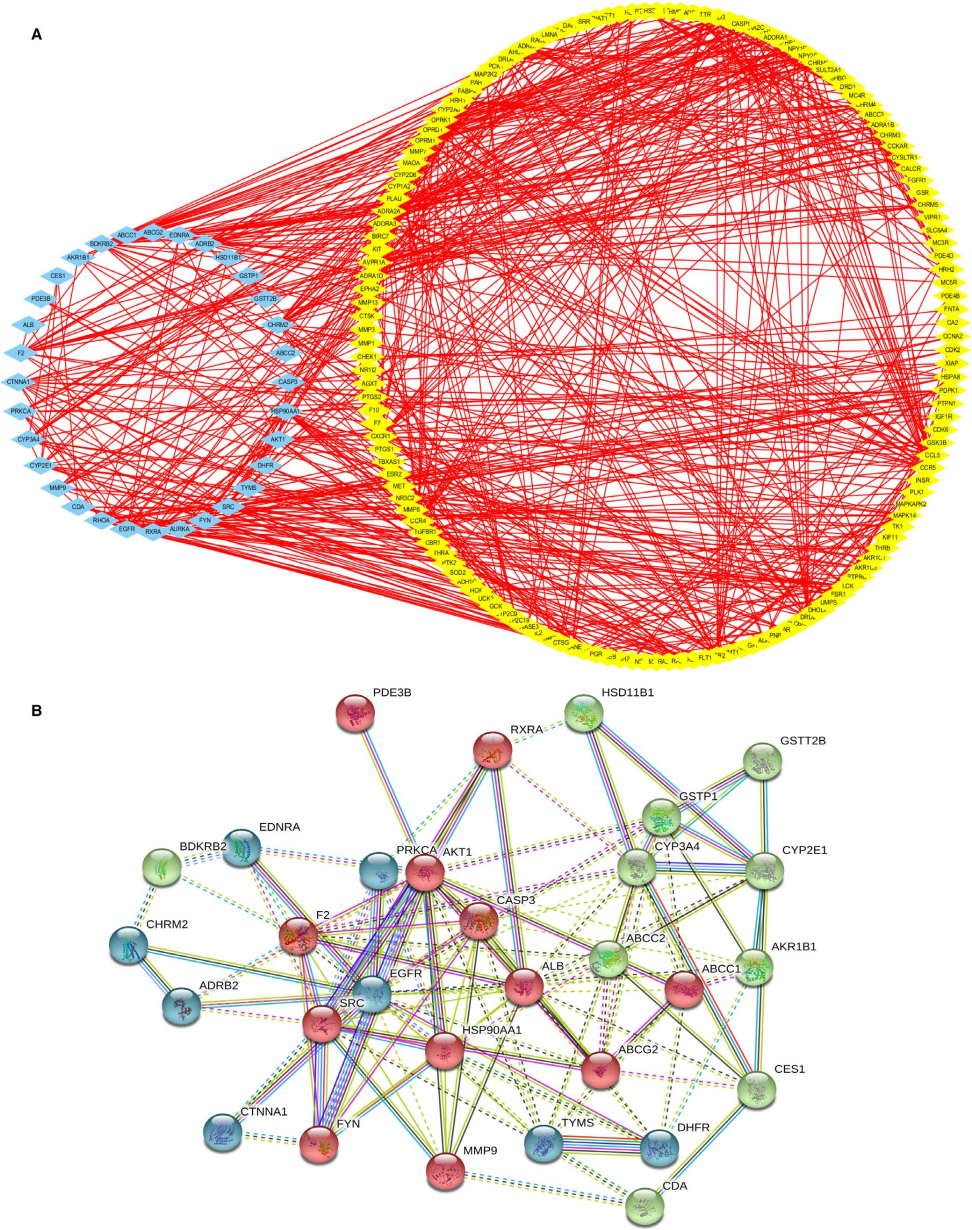
Protein interplay diagram of the predicted targets of 3TC. (A) Protein interplay diagram of the 269 predicted targets of 3TC. The blue nodes indicate the 32 core target genes of 3TC. (B) Protein interplay diagram of the 32 core targets of 3TC. Proteins interaction is mimicked by String 9.1. The networks showing similar functions are labelled with the same colour at the nodes. The line thickness of the edges connecting the nodes indicates the strength of data support. The proteins labelled at the red nodes were significantly associated with fluid shear stress and atherosclerosis, the green nodes were mainly associated with the metabolism of xenobiotics by the cytochrome P450 pathway, and the blue nodes were involved in both calcium and cAMP signalling pathways

The network pharmacology analysis revealed a total of 269 proteins as the predicted targets for 3TC with 164 identified by the Pharmmapper database and 105 by the ChEMBL database, respectively (Supplementary Table S3). The PPI network was constructed based on the target genes of 3TC using String database with a total of 32 core nodes in the network identified as the key targets of 3TC in the SAMP8 mice (Figure 5A). We used String 9.1 to mimic the interactions between key target proteins of 3TC to identify three groups of proteins with similar functions (Figure 5B).

### 3.6 Enrichment Analysis of the 3TC Key Target Networks

#### 3.6.1 GO Analysis

**FIGURE 6:**
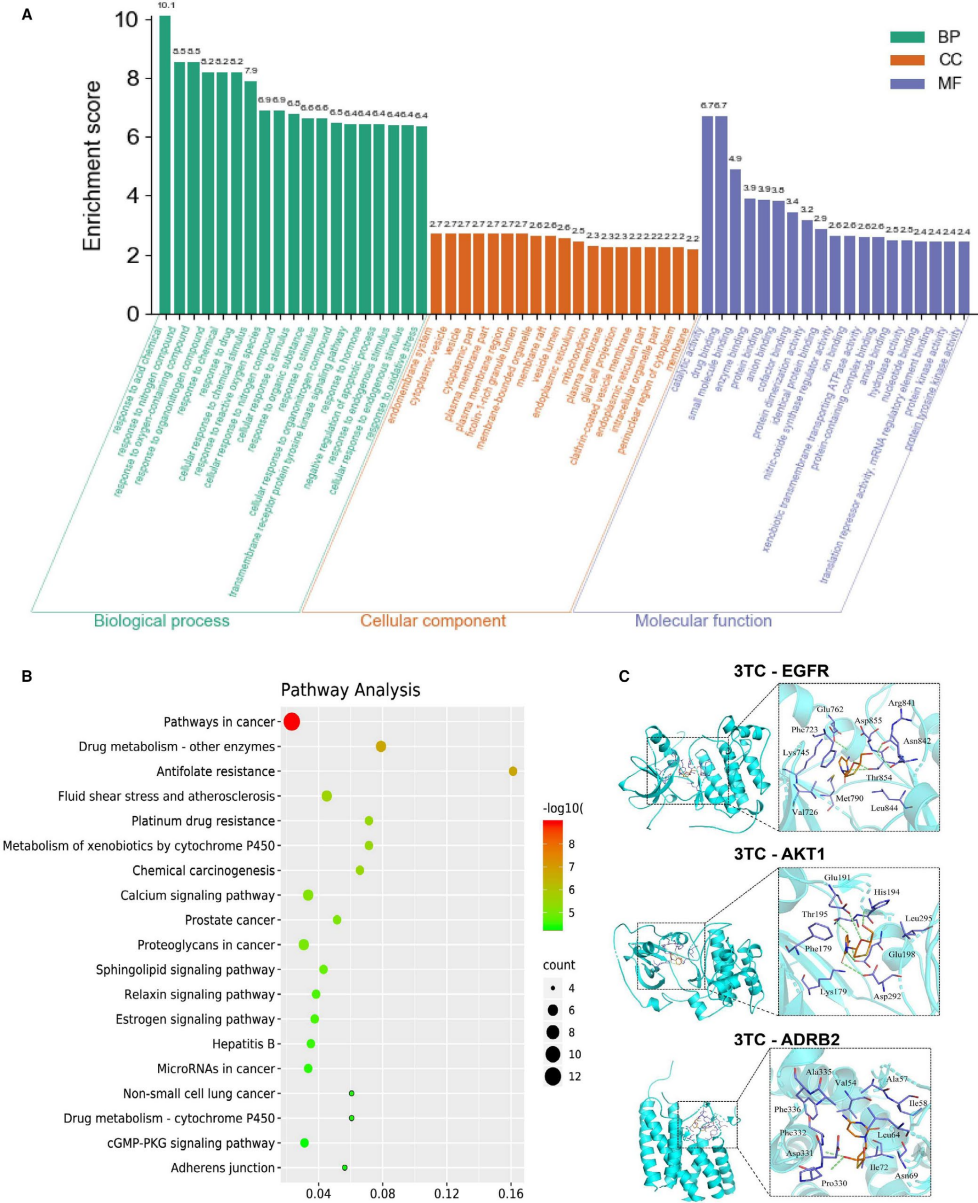
Gene Ontology (GO) enrichment and KEGG network analysis of target genes. (A) GO enrichment analysis of the 32 core target proteins of 3TC. The top 19 GO terms are presented in each of the three categories of GO terms (biological processes, cellular components and molecular functions). (B) The KEGG analysis of the 32 core targets of 3TC. The top 19 KEGG terms are presented. Bright red patches indicate high *p*‐values, and green patches indicate low *p*‐values. (C) Molecular models of 3TC binding to the predicted targets. Green labels indicate hydrogen bonds, small rods represent 3TC molecules, blue for amino acid residues, and orange for the ligand

The GO enrichment analysis of the target genes identified under the treatment of 3TC using STRING revealed a total of 550 biological processes with *p* < 0.05 (Figure 6A; Supplementary Table S4), such as the response to acid chemical, the response to reactive oxygen species and the negative regulation of apoptotic process.

The GO analysis also identified a total of 23 cellular components with *p* < 0.01, such as the endomembrane system, cytoplasmic vesicle, vesicle, cytoplasmic part and the plasma membrane part (Supplementary Table S5), while a total of 69 molecular functions identified by GO analysis were grouped in a total of 19 categories (Supplementary Table S6). It was worth noting that the tau protein binding term, which was thought to be linked to Alzheimer's disease, showed a significant relationship with 3TC (*p* < 0.05).

#### 3.6.2 Metabolic pathways of the 3TC target networks

A total of 109 significant treatment pathways were screened by KEGG analysis (*p* < 0.05; Figure 6B; Supplementary Table S7) with many pathways associated with cancers, including the pathways in cancer, chemical carcinogenesis, prostate cancer, proteoglycans in cancer and the microRNAs in cancer. Three pathways revealed to be related to neuroinflammation, cell death and neuronal signal transduction included the oestrogen signalling (*p* < 0.0001), the phosphoinositide 3‐kinase and protein kinase B (PI3K/Akt) (*p* < 0.0001), and the neuroactive ligand‐receptor interaction signalling (*p* < 0.001) pathways.^27^, ^28^, ^29^

#### 3.6.3 Binding mode

Docking studies were performed between 3TC and three selected potential targets, that is EGFR representing the oestrogen signalling pathway, AKT1 representing the P13K/Akt signalling pathway and ADRB2 representing the neuroactive ligand‐receptor interaction signalling pathway.

The results of binding mode analysis showed (Figure 6C) that the amino acid residues Thr854, Asn842, Arg841 and Glu762 of the receptor protein EGFR interacted with small molecules of 3TC ligands to form hydrogen bonds, while amino acid residues Lys55, Val40, Ala53, Met111, Val158, Asn114, Leu168, Ser155, Asp169, Lys153, Asn156s and Gly35 formed hydrophobic interactions with small molecules of the 3TC ligand. Amino acid residues Asp292, Glu191, His194, Glu198s and Thr195 of the receptor protein AKT1 formed hydrogen bond interaction with 3TC, while amino acid residues Phe179, Leu295, Glu198 and Lys179 formed hydrophobic interaction with 3TC. The amino acid residues Asp331 and Phe332 of ADRB2 interacted with 3TC to form a hydrogen bond, while the amino acid residues Phe336, Ala335, Val54, Ala57, Ile58, Leu64, Asn69, Ile72 and Pro330 of ABRB2 formed hydrophobic interaction with 3TC.

These results of docking studies provided evidence of 3TC binding to its targets, which was important to understand the mechanism of drug action. We further conducted in vitro experiments to verify the results of the bioinformatics analysis.

### 3.7 3TC protects HT22 cells from FA‐induced cytotoxicity by regulating a variety of molecules

**FIGURE 7:**
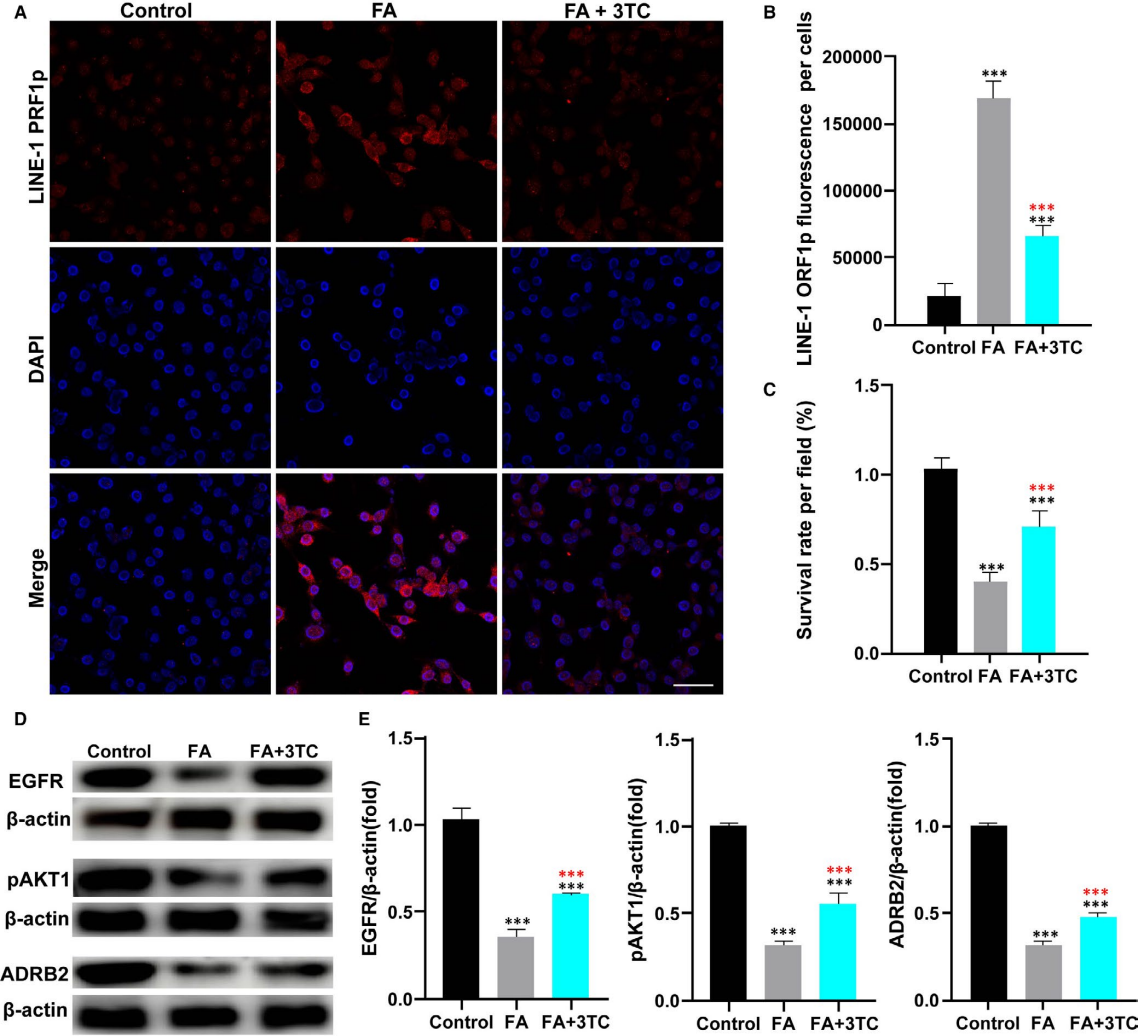
Experiments in vitro confirmed the key molecules of 3TC against neuronal degeneration. Senescence of mouse neuron HT22 cells is induced by formaldehyde (FA). (A) Immunofluorescence detection of LINE‐1 ORF1p in control, FA and FA+3TC groups (bar = 20 μm). (B) Quantification of immunofluorescence analysis in (A). (C) Quantification of surviving neurons per ×400 field in various groups. (D) EGFR, p‐AKT1 and ADRB2 were detected by Western blot analysis. (E) Quantification of Western blot analysis in (D). Student's *t*‐test: ***p < 0.001. Black asterisks represent comparison with the control group. Red asterisks represent comparison with the FA group

To explore the effects of 3TC as a reverse transcriptase inhibitor on senescent neurons in vitro, we induced the senescence in HT22 cells with FA. The results showed that LINE‐1 was activated in the senescent neurons, and this trend was reversed by 3TC (Figure 7A–B). In addition, 3TC showed an antagonistic effect on cell death induced by FA (Figure 7C).

As expected, stimulation of TH22 cells with FA for 24 h resulted in a significant inhibition in the expression of *EGFR*, *p‐AKT1* and *ADRB2* (*p* < 0.001), compared with an untreated control, while co‐treatment with 3TC led to a significantly increased expression of FA‐induced *EGFR, p‐AKT1* and *ADRB2* (*p* < 0.001; Figure 7D,E).

## 4 DISCUSSION

Cognitive declines, especially memory loss, are usually associated with natural ageing. The effects of ageing are particularly pronounced in the prefrontal cortex and hippocampus, the brain areas that are critically involved in cognition and mood.^30^ The SAMP8 mice were derived from the SAM‐P/2 line as a rapidly ageing mouse model of dementia. A previous study showed that the SAMP8 mice exhibited age‐related cognitive decline with a short lifespan, showing progressive learning and memory deficits as well as neuropathological hallmarks of dementia in the prefrontal cortex and hippocampus.^31^ The lifespan of SAMP8 mice is almost half of that of SAMR1, which were used as the controls for the SAMP8.^32^ In our study, the SAMP8 mice were selected as an ideal animal model to investigate the decline of cognitive function during ageing, with the homologous normally developing mice SAMR1 used as an anti‐senescence control group.

Ageing usually causes the body to lose weight due to bone wasting, muscle atrophy and dysfunction in the digestive system.^33^ Our results showed that 3TC partially alleviated the trend of weight loss. Compared with the untreated mice, the SAMP8 mice treated with 3TC showed a tendency to increase their body weight with age. These results are consistent with those reported previously, suggesting that 3TC improves the health of the bodies of the mice.^34^

In order to objectively and accurately evaluate the effects of 3TC treatment on improving the ageing characteristics of mice, we recorded the senescence scores during the treatment. Our results showed that with the increase of 3TC treatment time, SAMP8 mice showed a tendency to decelerate ageing. Specifically, the treatment of 3TC played a significant role in improving the glossiness of skin, the corneal opacity and the ulcer of the cornea caused by ageing in SAMP8 mice. These results suggest that the anti‐senescence target of 3TC exists on the skin and the corneal tissues.

Previous studies confirmed that L1 was used as a target for improving degenerative diseases caused by ageing, while reverse transcriptase inhibitors including 3TC were used as transposable inhibitors of L1.^13^ To date, the molecular mechanism of the role of L1 in the nervous system and the possibility of treating nervous system‐related diseases such as AD by regulating L1 have not been convincingly explained. Furthermore, studies have suggested that the genetic recombination in the APP gene may be a key event in the pathogenesis of sporadic AD, which is a type of dementia.^15^ The neuronal gene recombination has not been reported, while its occurrence and DNA damages and RNA intermediates require reverse transcriptase activities of protein functions. Therefore, the reverse transcriptase inhibitors such as 3TC may play a role in the prevention and treatment of AD which causes cognitive decline. This speculation was confirmed by our results of Morris water maze experiments investigating the effects of 3TC on cognitive ability in SAMP8 mice.

Our results of the Morris water maze tests showed that after 5 days of swimming training, SAMP8 mice having orally taken 3TC were able to find the hidden platform faster than untreated SAMP8 mice. Considering that the mice treated with 3TC showed larger bodyweight, which may be related to the amount of skeletal muscles, we recorded the swimming speed of each group of mice in order to eliminate the effect of muscle strength. Although the mice taking 3TC swam faster than other groups on the first day of swimming training, the difference did not persist in the next 4 days. Therefore, we rule out the influence of muscle strength on the results of the water maze tests. Similarly, the results of the space exploration experiment on the 6th day demonstrated that SAMP8 mice treated with 3TC showed a tendency to find the original platform more actively than untreated mice. These results suggest that 3TC treatment improves the cognitive and memory declines caused by ageing in SAMP8 mice.

To explore the potential molecular mechanisms of 3TC improving brain ageing, we applied real‐time quantitative PCR to measure the mRNA levels of candidate genes in hippocampal and cortical tissues of each group of mice. Our results suggested that 3TC treatment inhibited L1 induced by ageing in the brain and decreased the expression of genes related to IFN, SASP and AD, indicating that the signal pathways activated by ageing were suppressed by 3TC treatment. Our results suggest that 3TC may enhance intelligence and ageing phenotypes by inhibiting L1 transposition to ultimately improve the chronic inflammation in the brain caused by ageing. We note that more studies are needed to provide strong support for these molecular mechanisms of improving brain ageing by 3TC treatment revealed in this study. At present, the research on the pharmacological mechanism of reverse transcriptase inhibitors on nerve cells is being carried out in our laboratory using in vitro ageing glial and neuronal cell models.

Our results of HE and Nissl staining experiments indicated that 3TC remediated the neuron loss in hippocampus caused by ageing, ultimately ameliorating the neurodegeneration by rescuing neurons from death. Besides, we observed more apoptotic cells in the hippocampus of SAMP8 mice in comparison with SAMR1 mice. Our results further demonstrated that 3TC treatment significantly reduced the number of apoptotic cells, probably due to the direct effect of 3TC on the apoptotic pathway‐related proteins or other upstream molecules.

Learning and memory are important cognitive abilities that involve a complex network of functional brain regions working together to manage and process information. Therefore, any compounds showing effects on the brain should be taken cautiously with their potential targets fully understood. The network pharmacology approach is a systematic analytical technology used to explore the interaction networks of multiple factors such as diseases, drugs, genes and protein targets. We conducted the network pharmacology analyses, including PPI and enrichment analyses, based on a total of 269 potential targets (with 32 core targets) of 3TC annotated using Pharmmapper and ChEMBL databases. Our results demonstrated that 3TC may play important roles in maintaining the normal functions of the nervous system through both the oestrogen signalling and the PI3K/Akt and neuroactive ligand‐receptor interaction signalling pathways, which further explained the effects of 3TC on protecting the normal nerve functions and inhibiting the neuronal cell death. Representative molecules in the relevant pathways were selected to carry out molecular docking and in vitro experiments to confirm the results of bioinformatics analysis. In order to provide more evidence to support these speculated molecular mechanisms of 3TC working synergistically with the nervous system, further studies are necessary to identify the exact components involved and regulated in these pathways.

As a part of the antiretroviral therapy (ART), 3TC has been extensively studied in the past years and has been approved for the treatment of chronic hepatitis B virus (HBV).^35^ It must be noted that if 3TC is used to treat ageing‐related neurodegenerative lesions, it is necessary to consider its pharmacological toxicity due to the weak immune system in the elderly populations and cancer patients. Studies have shown that common adverse reactions of 3TC include upper respiratory tract infection, nausea, vomiting, abdominal pain and diarrhoea. Therefore, the clinical application of 3TC should be extremely cautious. At present, 3TC can be used as a pharmacological tool to study molecular mechanisms but not as a putative therapy.

In summary, our study has demonstrated for the first time that 3TC improves the cognitive functions of SAMP8 mice by reversing the ageing of the brain, providing experimental evidence for the use of reverse transcriptase inhibitors as the potential treatments for neurodegenerative diseases.

## CONFLICT OF INTEREST

The authors declare that they have no conflict of interest.

## AUTHOR CONTRIBUTIONS

**Ming Li:** Data curation (equal); Methodology (equal); Writing‐original draft (equal); Writing‐review & editing (equal). **Jie Zhao:** Methodology (equal). **Qi Tang:** Methodology (equal). **Qingchen Zhang:** Project administration (equal). **Yong Wang:** Methodology (equal). **Jian Zhang:** Project administration (equal). **Yingying Hao:** Project administration (equal). **Xiaohui Bai:** Data curation (equal); Writing‐review & editing (equal). **Zhiming Lu:** Data curation (equal); Methodology (equal).

## Supporting information

Table S1

Table S2

Table S3

Table S4

Table S5

Table S6

Table S7

## Data Availability

The data that support the findings of this study are available from the corresponding authors upon reasonable request.
